# Shift-field refinement of macromolecular atomic models

**DOI:** 10.1107/S2059798320013170

**Published:** 2020-11-19

**Authors:** K. Cowtan, S. Metcalfe, P. Bond

**Affiliations:** a Department of Chemistry, University of York, York, United Kingdom; b Derpartment of Mechanical Engineering, McGill University, Montréal, Canada

**Keywords:** refinement, low resolution, computational methods

## Abstract

The aim of crystallographic structure solution is typically to determine an atomic model which accurately accounts for an observed diffraction pattern. A key step in this process is the refinement of the parameters of an initial model, which is most often determined by molecular replacement using another structure which is broadly similar to the structure of interest. In macromolecular crystallography, the resolution of the data is typically insufficient to determine the positional and uncertainty parameters for each individual atom, and so stereochemical information is used to supplement the observational data. Here, a new approach to refinement is evaluated in which a ‘shift field’ is determined which describes changes to model parameters affecting whole regions of the model rather than individual atoms only, with the size of the affected region being a key parameter of the calculation which can be changed in accordance with the resolution of the data. It is demonstrated that this approach can improve the radius of convergence of the refinement calculation while also dramatically reducing the calculation time.

## Abstract 2

Shift-field refinement is an approach to the refinement of a model against a set of structure-factor observations which is not tied to an underlying atomic parameterization. The performance of the method is evaluated for the refinement of positional parameters over a set of 452 molecular-replacement problems.

## 1. Introduction   

Crystallographic refinement, in which the parameters of an atomic model are optimized to best explain an observed diffraction pattern, is an important stage in the structure-solution process. In addition to improving the correspondence between the atomic model and the scattering matter of the crystal, the refinement step typically incorporates the calculation of statistics such as the *R* factor and free *R* factor (Brünger, 1992 ▸), which provide a measure of reliability for the resulting model.

Crystallographic refinement has traditionally involved the optimization of positional, thermal and other parameters that describe each individual atom in the crystal structure (Driessen *et al.*, 1989 ▸). In the case of macromolecular refinement, disorder and thermal motion limit the resolution of the diffraction pattern and thus the number of available observations; this means that the data are insufficient to allow us to uniquely determine all of the atomic parameters. Geometrical restraints on the stereochemistry are therefore incorporated in order to ensure that the refinement equations are overdetermined. The diffraction observations and stereochemical restraints introduce very different patterns of correlation among model parameters: each diffraction observation impacts *every* positional coordinate, whereas any given geometric restraint affects only a small number of atoms, introducing strong correlations between the coordinate parameters of those atoms. The use of stereochemical restraints therefore leads to a substantial increase in the complexity of the topology of the refinement landscape, leading to slower convergence; this is only partially mitigated by use of higher order derivatives of the target function (Murshudov *et al.*, 2011 ▸).

An alternative approach to this problem is to reduce the number of model parameters to better reflect the amount of information that is present in the data. In particular, if the fine-scale features of the model (including the desired bond lengths and angles, but also less conserved features such as side-chain rotamers) are assumed to be correct, then coarse-scale shifts can be applied to the model in order to better account for features of the diffraction pattern, for example domain motions or differences in chain placement between a close structural homologue and the experimental target. Terwilliger *et al.* (2013 ▸) proposed a method for doing this by performing a local search for electron-density features which match a given model chain fragment for consecutive fragments extracted from the atomic model. Other approaches explored in the field of cryo-electron microscopy include the use of normal-mode analysis to explore difference in model conformation (Tama *et al.*, 2004 ▸; Suhre *et al.*, 2006 ▸) and the hierarchical refinement of rigid model domains (Joseph *et al.*, 2016 ▸).

An alternative approach was proposed by Cowtan & Agirre (2018 ▸) in which parameter shifts are calculated, based on the modification of existing refinement algorithms, which are applicable to arbitrarily sized regions of the map rather than to individual atoms only. A preliminary test demonstrated the ability of this approach to refine isotropic thermal parameters for an atomic model given data to limited resolution, and is now available as a tool for *B*-factor refinement in the *Coot* model-building software (Emsley & Cowtan, 2004 ▸).

The advantage of seeking coarse-scale model shifts to explain the observations is that the resulting shifts largely preserve the fine-scale features of the model (such as bond lengths) and can therefore be performed without the introduction of stereochemical restraints. The simplicity of the refinement landscape, coupled with the ability to refine at low resolution, ultimately leads to faster calculations and faster convergence.

### 1.1. Crystallographic refinement   

The crystallographic refinement calculation is characterized by optimization of the parameters of the atomic model which, in most implementations, involves trying to minimize the features of the difference map that arises from the disagreement between the observed and calculated structure factors (Driessen *et al.*, 1989 ▸; Sheldrick & Schneider, 1997 ▸; Henderson & Moffat, 1971 ▸). In modern implementations, refinement calculations include likelihood weighting to make optimal use of the information available (Read, 1986 ▸; Murshudov *et al.*, 1997 ▸; Blanc *et al.*, 2004 ▸; Afonine *et al.*, 2012 ▸).

The disagreement between the model and the data may be expressed in reciprocal space as differences between observed and calculated structure factors or in real space though a difference map calculated from the structure-factor differences, with the two formulations being mathematically indistinguishable (Agarwal *et al.*, 1981 ▸; Bricogne, 2001 ▸). In the Lifchitz formulation, the calculated electron-density map is differentiated with respect to the (positional or thermal) parameters of interest (Bricogne, 2001 ▸); a positive correlation between this gradient map and the difference map, when integrated over the volume of an atom at a given position in the map, implies that the agreement between the model and observations can be improved by modifying the corresponding parameters of the atom at that position.

The refinement of atomic coordinates converges quickly when the current atomic model coordinates are close to the true values (assuming that the model is in all other respects a good description of the crystal cell) but often fails to converge to a global minimum when parts of the initial model are far from the true coordinates: this is often the case when starting from a molecular-replacement model which shares only modest sequence identity with the structure of interest.

### 1.2. Shift-field refinement   

Cowtan & Agirre (2018 ▸) made two modifications to this approach. Firstly, instead of integrating over the volume of a single atom, the agreement between the gradient map and the difference map is integrated over a much larger spherical region whose volume is determined by the resolution of the data. Secondly, agreement is determined by linear regression, where the gradient maps with respect to each type of model parameter are used as predictors of the difference map. This regression calculation, which is performed via Fourier transforms (Bricogne, 2001 ▸), produces a spatial field of shifts for each model parameter and is referred to as a *shift field*.

Shift-field refinement therefore attempts to account for features of the difference map through adjustments to the calculated electron-density map, which may or may not be determined from a model. These adjustments, which may include moving the density (for example by moving atoms) or changing the density peak heights (for example by adjusting *B* factors), are described by the following system of equations,
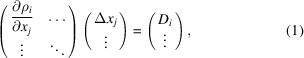
where *x_j_* is some parameter of the electron density (such as the position of a grid point in the map), Δ*x_j_* is the shift to be applied to that parameter, ρ_*i*_ is a calculated electron-density value determined from the current model through a position in the map close to *x_j_*, and *D_i_* is the difference-map value at that position in the map. In order to determine the coordinate shifts to be applied to the model density at any point in the map without the calculation becoming underdetermined, we accumulate information from a spherical region of many electron-density points (denoted by the index *j*) about the point denoted by index *i*. By choosing a large enough sphere (and therefore a large enough number of electron-density points), the problem is always well determined and can be solved by least-squares or, better, by weighted linear least-squares regression in which density values closer to the point denoted by the index *i* are given greater weight (Cowtan & Agirre, 2018 ▸). In addition to the conventional parameters *x*, *y*, *z* and *B*, an additional constant term can be included in the regression calculation to mop up a roughly constant offset between the model density and the observations, which may arise from errors in low-resolution terms.

The method as described performs a single step of shift-field refinement. As in existing refinement strategies, the calculation does not typically converge in a single cycle and so the calculation must be applied iteratively. At each step, a new model electron-density map ρ is calculated, a likelihood-weighted difference map *D* is determined using the observed structure-factor amplitudes and a shift field is determined. The initial stages of the calculation are performed at low resolution to allow large shifts to whole domains and the resolution limit is increased at each step.

### 1.3. Comparison to previous work   

The method presented here is essentially the same as the proof-of-concept calculation of Cowtan & Agirre (2018 ▸), except that we now explore the application of the method to coordinate rather than *B*-factor refinement and test whether the inclusion of an additional constant parameter provides any benefits. We have introduced the term ‘shift-field refinement’ in this paper to distinguish the method described here from conventional refinement calculations and to highlight the applicability of the method to the refinement of a map against observations, although we do not utilize this feature in the current work.

While shift-field refinement addresses a similar problem to the model-morphing approach of Terwilliger *et al.* (2013 ▸), there are significant underlying differences. Terwilliger and coworkers calculate refinement gradients for individual atoms based on their local environment and then apply a moving average of the shifts along the chain, whereas shift-field refinement averages shifts over a spherical region around each map grid point without reference to a model. On theoretical grounds, we would therefore expect the two methods to be complementary: model morphing should be capable of sliding one chain longitudinally with respect to a neighbouring chain because shifts are only propagated along chains, while shift-field refinement should be capable of capturing large domain shifts because the shifts can capture information from neighbouring chains, and do so more quickly by working at lower resolution and not requiring geometrical restraints.

## 2. Methods   

To evaluate the effectiveness of shift-field refinement for atomic coordinates, refinement was attempted using 452 molecular-replacement problems from a set compiled by Bond *et al.* (2020 ▸). Bond and coworkers identified 1351 well refined structures from the Protein Data Bank (Berman *et al.*, 2007 ▸) for which experimental data were available, which uniformly sample the resolution range 1.0–3.5 Å. The structures were selected at random, but had to be good quality as judged by five validation metrics: *DCC* *R*_free_, clashscore, Ramachandran outliers, side-chain outliers and RSRZ outliers. They also had to be diverse, containing no protein chains with a sequence identity of 50% or more. Bond *et al.* (2020 ▸) also produced molecular-replacement models which lead to initial maps that sample a range of map qualities. They performed sequence alignment of the search model on the deposited model using *GESAMT* (Krissinel, 2012 ▸) before molecular replacement (MR) using *Sculptor* and *Phaser* (Bunkóczi & Read, 2011 ▸) and a short conventional refinement using *REFMAC*5. For many of these structures, either MR did not successfully find all copies of the molecule, some copies of the molecule were incorrectly positioned or multiple chain sequences were present in the structure, so this set of structures was reduced to a subset for which there was only one chain in both the MR structure and the deposited structure, leaving a total of 452 models. The free *R* factors for the models, after preliminary refinement against the deposited reflection data, range from 22 to 56%. The target structures, search models and relevant quality metrics are listed in the supporting information to this paper.

Each model was then refined using one of the following four procedures. The choices of parameters for these procedures will be discussed below.

(i) 20 cycles of conventional refinement (*i.e.* refinement of individual atomic coordinate and isotropic *B* factors using a maximum-likelihood target) in *REFMAC*5 version 5.8.258 from *CCP*4 version 7.1 (Kovalevskiy *et al.*, 2018 ▸).
(ii) 200 cycles of jelly-body refinement in *REFMAC*5 followed by 20 cycles of conventional refinement in *REFMAC*5. Jelly-body refinement is a method developed specifically for the refinement of coarse-grained shifts to improve the radius of convergence of refinement with limited resolution data (Murshudov *et al.*, 2011 ▸).
(iii) 12 cycles of shift-field refinement of positional (*x*, *y* and *z*) parameters starting at a resolution of 6 Å and increasing stepwise to 3 Å, followed by 20 cycles of conventional refinement in *REFMAC*5. The radius of the shift-field regression sphere was set to four times the resolution of the current cycle. Refinement at lower resolution and with a larger regression sphere leads to a larger radius of convergence but limits the ability of the method to refine detailed features (Cowtan & Agirre, 2018 ▸).
(iv) 12 cycles of shift-field refinement followed by 20 cycles of conventional refinement (as in the previous procedure) but also including the additional constant term in the linear regression calculation. The inclusion of the constant term is an additional feature that is not present in traditional refinement which may or may not contribute to the performance of the shift-field calculation.

The results and computational requirements of a refinement calculation are influenced by the choice of program parameters and the number of cycles run. *REFMAC*5 uses data to the resolution limit by default and runs for a user-determined number of cycles rather than using a convergence criterion. The conventional refinement calculation is performed here using the command-line defaults, with the exception of the number of cycles, which was increased from the *CCP*4 graphical user interface default of 10 (Potterton *et al.*, 2003 ▸) to 20 on the basis of tests with automated model building. For jelly-body refinement the default parameters were taken from the *CCP*4 graphical user interface, and the number of cycles was set to 200 to address the slower convergence of this method (Kovalevskiy *et al.*, 2018 ▸); inspection of the refinement statistics with cycle number suggests that the refinement has essentially converged by the end of the computation. For shift-field refinement the resolution and number of cycles were determined by a coarse search to optimize the results using a smaller data set; we found that running for significantly more than 12 cycles can distort the model geometry to the point where the final conventional refinement cannot restore it.

### 2.1. Metrics for evaluating refinement results   

In order to evaluate the behaviour of the different refinement protocols, quality metrics are required. Given the size of the test set, these must be quantitative and not require manual evaluation on a per-structure basis. We consider two types of metrics: those which depend on some estimate of the ‘true’ structure (which we refer to as extrinsic metrics) and those which do not (which we refer to as intrinsic metrics).

We calculate extrinsic metrics based on the structure deposited in the PDB (Berman *et al.*, 2007 ▸). Common metrics include coordinate differences and phase errors or map correlations. Given that the aim of this work is to improve the preliminary refinement of MR models, we assume that the deposited structures are a better description of the data than the refined search models and will use C^α^-atom r.m.s.d. to evaluate main-chain fit. This does depend on the deposited model being largely correct, and on establishing which atoms of the MR model are structurally homologous to corresponding atoms in the true structure.

Intrinsic metrics include the crystallographic *R* factors and model geometry metrics such as bond-length variability, Ramachandran and rotamer outliers and clashscore. The agreement between observed and calculated structure-factor amplitudes is particularly useful because the residual error in the calculated complex structure factor is typically independent in phase from the structure factor itself, and thus the error in the amplitude provides an intrinsic estimator of phase error (Srinivasan & Parthasarathy, 1976 ▸; Murshudov *et al.*, 1997 ▸). This assumption is however biased by refinement of the model against the structure-factor amplitudes, which can be mitigated by use of the crystallographic free *R* factor (Brünger, 1992 ▸; Lunin & Skovoroda, 1995 ▸), calculated in this case by *REFMAC*5 (Murshudov *et al.*, 2011 ▸). The utility of the free rather than work *R* factor in this context is also supported by comparison with the phase errors (Supplementary Table S2). We also evaluate geometry statistics determined by *REFMAC*5 and *MolProbity* (Chen *et al.*, 2010 ▸). These metrics are calculated for each of the 452 test structures after refinement of the MR solutions using each of the four refinement procedures outlined above.

### 2.2. Shift-field implementation   

The mathematical details of the shift-field method have been described by Cowtan & Agirre (2018 ▸); however, in order to facilitate the reimplementation of the method by other authors we outline it in more detail here. The steps of the calculation are as follows.

(i) Read in the model to be refined.
(ii) Read in the structure-factor amplitudes and standard deviations along with the associated free *R* flags.
(iii) Repeat the following steps a user-specified number of times (in this case 12).
  (1) Determine the resolution for the current cycle (in this case linearly increasing with cycle number from 6 to 3 Å).
  (2) Determine the regression-sphere radius for the current cycle (in this case four times the resolution).
  (3) Calculate the structure factors and phases (with bulk-solvent correction) for the current model at the current resolution.
  (4) Determine σ_A_ coefficients (Read, 1986 ▸) for the calculated phases using the method of Cowtan (2008 ▸) and use them to calculate difference map coefficients.
  (5) Calculate an electron-density map ρ from the calculated structure factors and a difference map *D* from the weighted difference-map coefficients.
  (6) Calculate the derivatives ∂ρ/∂*x_i_* of the calculated electron-density map ρ with respect to each of the parameters *x_i_* to be refined (in this case, the fractional coordinates *u*, *v* and *w*).
  (7) Calculate a 3-vector of electron-density maps (the residual vector) from the product of the difference map and the three gradient maps *D*(∂ρ/∂*x_i_*).
  (8) Calculate a 3 × 3 symmetric matrix (the normal matrix) of electron-density maps from the product of the gradient maps with each other (∂ρ/∂*x_i_*)(∂ρ/∂*x_j_*).
  (9) Smooth each of the maps in the residual vector and normal matrix by convolution with the function *f*(*r*) = 1 − (*r*/*r*_0_)^2^ for *r* < *r*_0_, where *r*_0_ is the current regression radius.
  (10) Solve for the shift field, which, for each grid point in the map, is the vector of parameter shifts Δ*x_j_* that when pre-multiplied by the normal matrix gives the residual vector (taking the values at the grid point).
  (11) Loop over all atoms and update their fractional coordinates by the values of the shift-field maps interpolated to the current coordinates of the atom.
(iv) Write out the atomic model with the updated parameters.

A simplified process diagram is shown in Supplementary Fig. S3. The whole procedure has been implemented in C++ using the Clipper crystallographic libraries (Cowtan, 2003 ▸).

## 3. Results   

**Figure 1:**
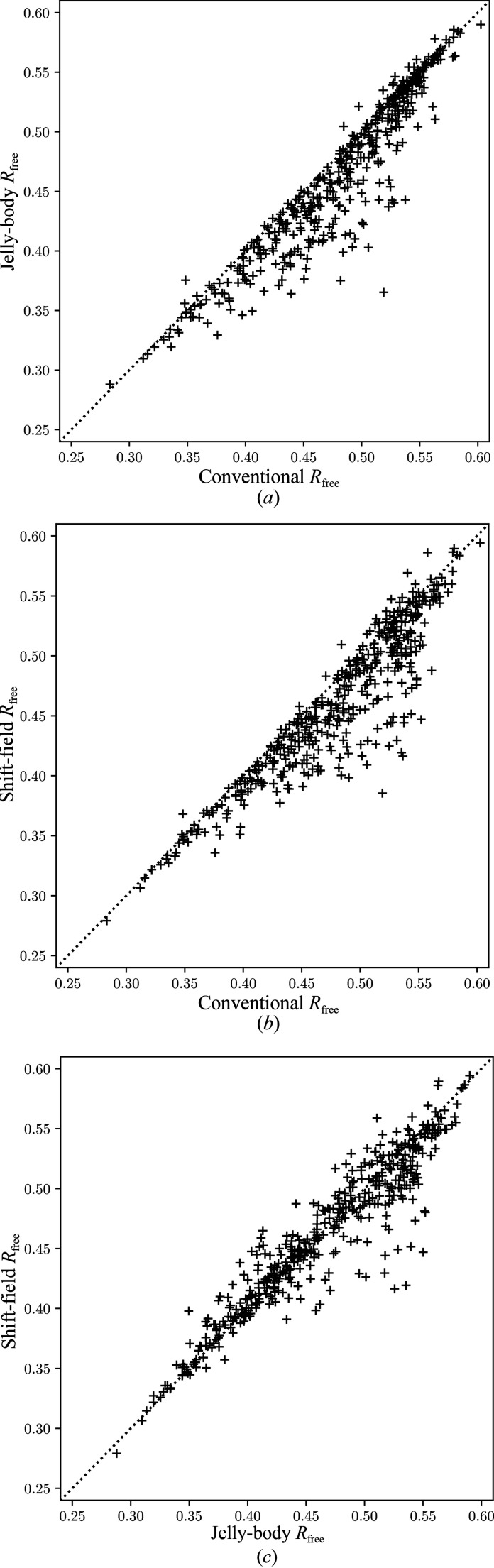
Comparison of the free *R* factor after the various refinement procedures. (*a*) Jelly-body refinement compared with conventional refinement alone. (*b*) Shift-field refinement compared with conventional refinement alone. (*c*) Shift-field refinement compared with jelly-body refinement. Points below the diagonal indicate a better result for the method on the *y* axis.

Each of the four refinement procedures described above were applied to each of the 452 molecular-replacement models in turn. Fig. 1 ▸ shows a comparison between the final free *R* factors for models refined using conventional refinement alone, jelly-body plus conventional refinement and shift-field plus conventional refinement (with the omission of the constant term). Fig. 1 ▸(*a*) compares conventional with jelly-body refinement. In the majority of cases, the inclusion of jelly-body refinement causes a very small reduction in the free *R* factor compared with conventional refinement alone. However, there are a subset of cases where the reduction in free *R* factor is far more substantial, with the extreme case being PDB entry 4c2q, where the inclusion of jelly-body refinement leads to a reduction in the free *R* factor of 15%. Fig. 1 ▸(*b*) compares conventional refinement and shift-field refinement, with the results showing a similar pattern of improvement to the jelly-body method. The largest improvement is again for PDB entry 4c2q, with an improvement in free *R* factor of 13%. Fig. 1 ▸(*c*) compares jelly-body and shift-field refinement, confirming that while there are outliers for which either refinement method may lead to a better reduction in free *R* factor, shift-field refinement provides the lowest free *R* factor for a larger number of test cases and offers the greatest benefits for the challenging cases where the free *R* factor is highest. Comparison of the *R* factors for the work rather than free sets yields similar results (see supporting information).

The MR search models often represent only part of the structure owing to sequence nonhomology, pruning, omission of waters and ligands, and in some cases significant conformational differences, and as a result the *R* factors for the refined MR models are substantially higher than the deposited structures (an average of 42% versus 17%). This shows that the benefits of shift-field refinement are not contingent on having a substantially complete model.

**Figure 2:**
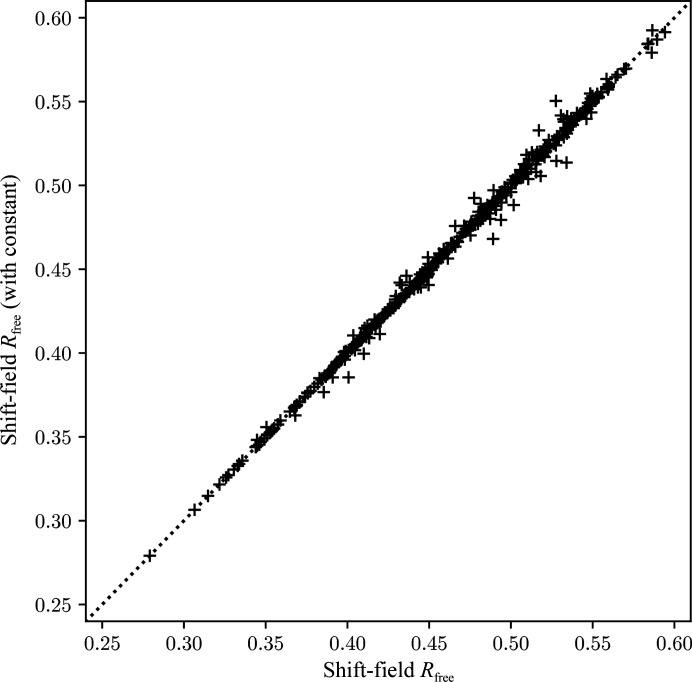
Comparison of the free *R* factor after shift-field plus conventional refinement with the shift-field regression calculation either including (*y* axis) or omitting (*x* axis) the constant term.

Fig. 2 ▸ shows a comparison of free *R* factor after shift-field refinement plus conventional refinement where the constant term is either omitted or included. Inclusion of the constant term appears to offer no significant benefit yet significantly increases the number of fast Fourier transforms required, so it is not recommended. We hypothesize that the principal benefit of including the constant term would be to address the case of missing low-resolution reflections which lead to long-range ripples across the map. However, these are already removed in practice by the omission of these terms from the difference-map calculation when using maximum-likelihood difference map coefficients (Murshudov *et al.*, 2011 ▸).

Geometry validation indicators for models from the various refinement procedures are given in Table 1 ▸. The differences are minor and there is no clear pattern; for example, jelly-body refinement produces the lowest proportion of Ramachandran outliers but the highest proportion of rotamer outliers.

We also examined the stability of the shift-field method when applied to the deposited structures in order to determine the extent to which the method is able to preserve the features of the most correct model available. Shift-field refinement is performed with no stereochemical restraints, and so it is possible that the model may be significantly distorted or overfitted. For each case, the deposited structure was subjected to 20 cycles of refinement in *REFMAC*5 and a zero-cycle run was then used to evaluate the refinement statistics. 12 cycles of shift-field refinement were applied to the resulting model, and another zero-cycle run was used to determine the refinement statistics without performing any further conventional refinement. Application of shift-field refinement caused an increase in the average of the crystallographic *R* factor over the 452 test cases from 17.3% to 18.5%, while the free *R* factor increased from 20.7% to 21.8%. The results show no evidence of overfitting (since the free *R* factor increases by no more than the work *R* factor), and the models suffer only minor distortions, which are small in comparison to the convergence radius of conventional refinement.

**Figure 3:**
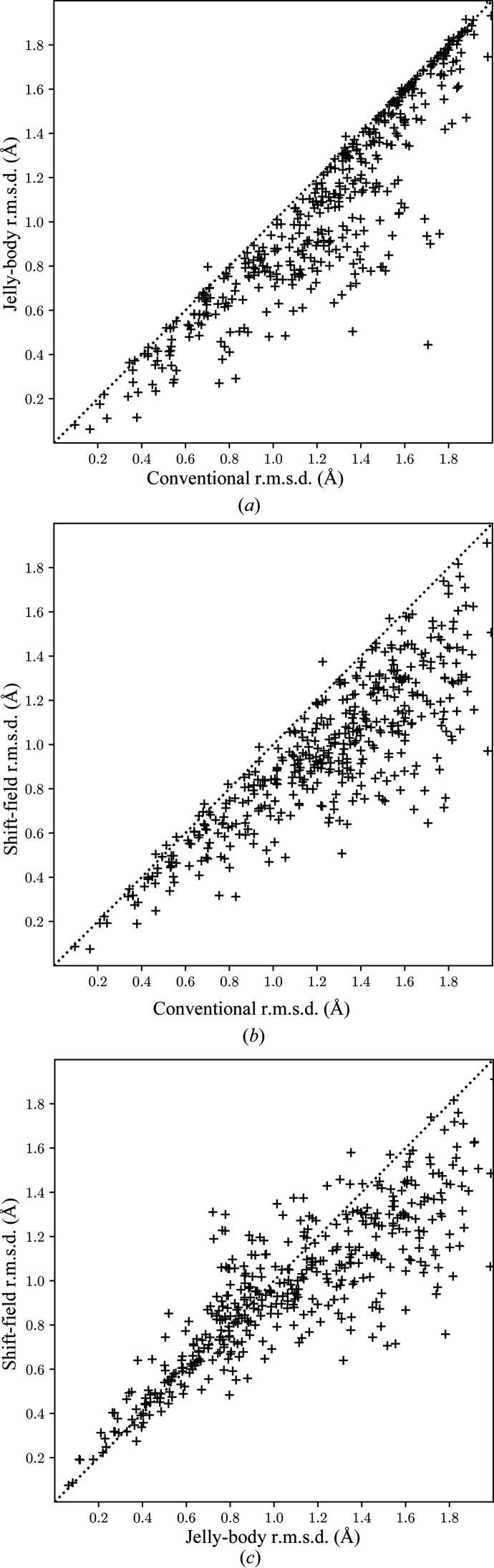
Comparison of the root-mean-square difference in C^α^ coordinate positions between matched residues of the refined molecular-replacement model and the refined deposited structure under different refinement protocols. (*a*) Jelly-body refinement compared with conventional refinement alone. (*b*) Shift-field refinement compared with conventional refinement alone. (*c*) Shift-field refinement compared with jelly-body refinement. Points below the diagonal indicate a better result for the method on the *y* axis.

Fig. 3 ▸ shows comparisons between the same methods as in Fig. 1 ▸ but for the root-mean-squared C^α^ coordinate difference (r.m.s.d.) in comparable regions of the refined and deposited models. To ensure a valid comparison, the coordinate differences must be calculated over the same subset of atoms for each case, but the MR models may have substantial insertions and deletions compared with the target structure. Therefore, a list of comparable residues was first identified for each case by performing a sequence alignment between the MR and deposited models. We then select from the successfully aligned residues those for which the C^α^ atom of any one of the refined models lies within 3 Å of the deposited coordinate in order to exclude outlier residues which otherwise dominate the r.m.s.d. Fig. 3 ▸ shows a very similar pattern to Fig. 1 ▸, with both jelly-body refinement and shift-field refinement leading to an improvement in the r.m.s.d. of the deposited model, but with shift-field refinement generally offering larger benefits for the poorest models.

**Figure 4:**
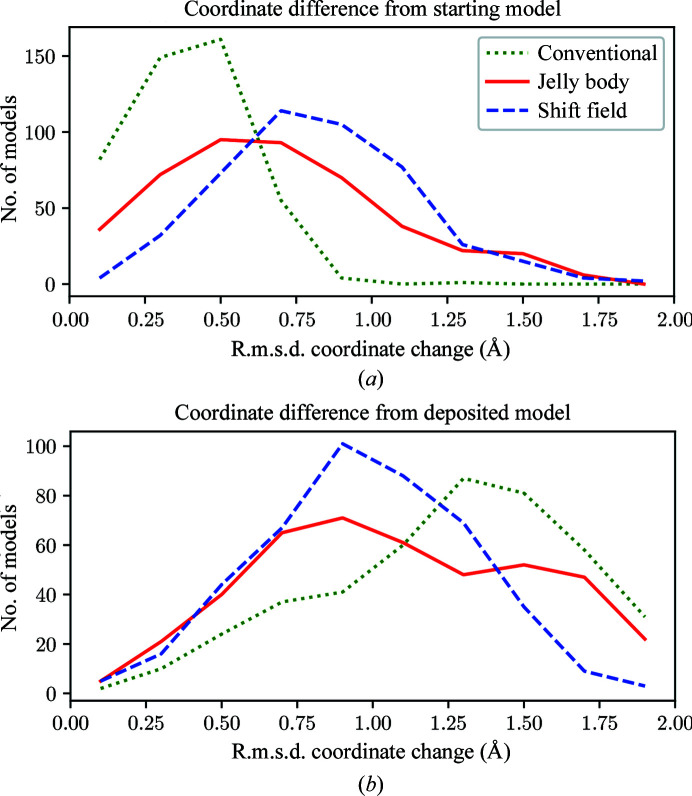
Comparison of the distribution of r.m.s.d. values over the 452 test structures between the initial model and models refined using the conventional, jelly-body and shift-field refinement protocols (*a*) and between the refined models and the deposited structure (*b*). The distributions are binned in steps of 0.2 Å but are plotted with lines for ease of comparison.

Fig. 4 ▸(*a*) shows the distribution of r.m.s.d.s between each of the 452 initial MR models and models refined by conventional, jelly-body or shift-field refinement. Jelly-body refinement moves the initial model substantially further than conventional refinement, with shift-field refinement moving the model further still. Fig. 4 ▸(*b*) shows a similar comparison of all of the models with the corresponding deposited structure. The conventionally refined models are on average the furthest from the deposited structure and the shift-field-refined models are closest, which implies that the larger movements shown in Fig. 4 ▸(*a*) primarily move the models towards the deposited structures.

**Figure 5:**
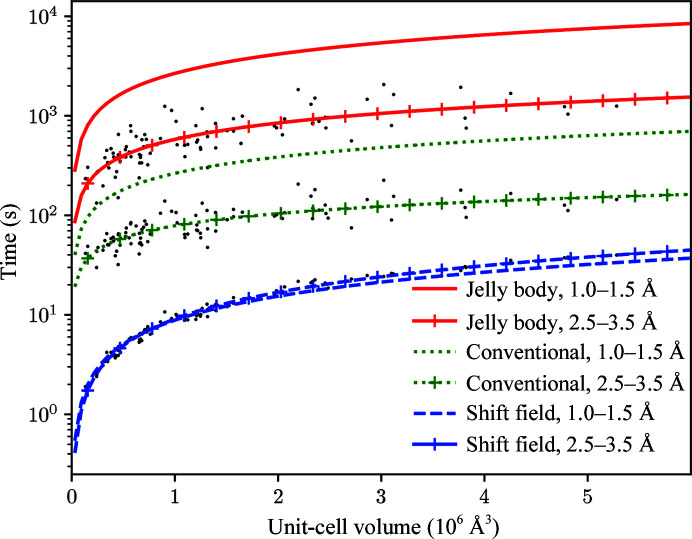
Comparative CPU times (in seconds) for the three refinement protocols averaged over 160 structures between 1.0 and 1.5 Å resolution and 90 structures between 2.5 and 3.5 Å resolution. Results are given for shift-field refinement alone (without the conventional refinement step), for 200 cycles of jelly-body refinement in *REFMAC*5 and for 20 cycles of conventional refinement in *REFMAC*5. The least-squares linear fit of time against unit-cell volume is shown for each method and resolution range, with scatter points shown for the low-resolution subsets.

The variations in calculation time as a function of unit-cell volume for shift-field and jelly-body computations are shown in Fig. 5 ▸ for high-resolution (better than 1.5 Å) and low-resolution (worse than 2.5 Å) subsets of the data. In all cases the calculation becomes slower with increasing structure size. For conventional and jelly-body refinement, higher data resolutions lead to longer calculation times. Since in these tests the shift-field calculation is performed at the same resolution for each case, the calculation time is independent of resolution. For the least favourable case of a large (6 × 10^6^ Å^3^) unit cell and low (3 Å) resolution, the shift-field calculation is still four times faster than conventional refinement and 40 times faster than jelly-body refinement, with these numbers increasing by a further factor of five for higher resolution data sets. The additional time penalty for jelly-body refinement arises primarily from slower convergence and thus a greater number of required cycles, while the speed of the shift-field calculation arises in part from being able to perform the calculation at lower resolution, varying from 6 to 3 Å over the course of the calculation. The disparities with conventional and jelly-body refinement can be somewhat reduced by reducing the number of *REFMAC*5 cycles; for example, Kovalevskiy *et al.* (2018 ▸) use only 100 cycles of jelly-body refinement in favourable cases. Further large-scale testing is required to optimize computational cost against model quality for all of the procedures.

We further investigated the disparities in computational cost by performing refinement calculations using both *REFMAC*5 and shift-field refinement at the same resolution (3.5 Å) for each structure. In this case the per-cycle calculation time is about equal for the two methods (about 1 s per cycle using our current hardware), confirming that the ability to run at lower resolution and for fewer cycles accounts for almost all of the difference in computation. Startup time also plays a role, being negligible for shift-field refinement but an average of 30 s for *REFMAC*5. The similar per-cycle costs at comparable resolutions are unsurprising since both approaches to refinement require an electron-density calculation and a few tens of fast Fourier transforms, although the shift-field algorithm may be amenable to some optimization. Despite this similarity, the numbers of density grid points which contribute to gradient determination at a given atom or grid point are very different: in the case of conventional refinement the contributing volume covers the volume of an atom: usually of the order of ten grid points for an individual atom in the case of 3 Å resolution data and thus a 1 Å grid. In the case of shift-field refinement, the gradient at a point in a 3 Å resolution map will be determined using all of the density points in a sphere of 12 Å radius: more than 5000 grid points.

When developing software tools for unknown problems, our principal focus is on the results of the automatic application of the method to large systematically chosen ensembles of test structures because this provides the most useful predictor of the distribution of results that a nonspecialist user might obtain when applying the method using default options to an unknown structure. Nonetheless, it is occasionally possible to obtain insights into the functioning of different methods by examining individual models. We examined cases for which either shift-field or jelly-body refinement substantially outperformed the other method and highlight two such examples below.

**Figure 6:**
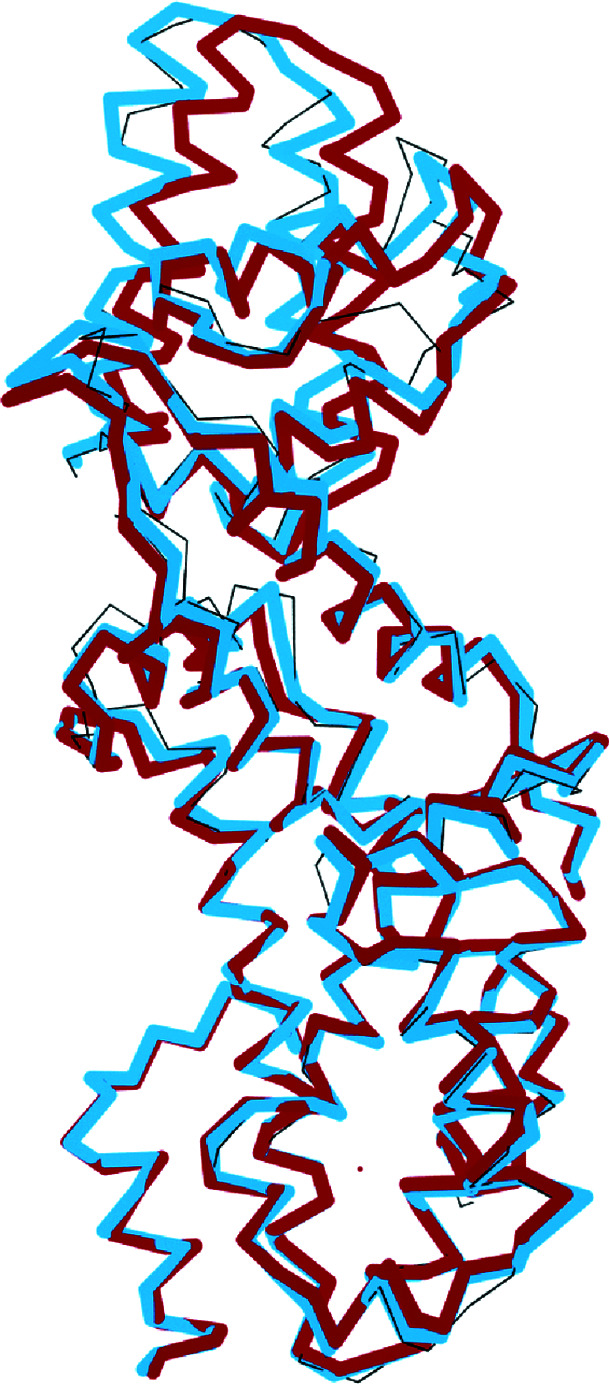
Comparison of a section through models for PDB entry 4l9m, showing the C^α^ trace of the jelly-body refined model (thick dark bonds), the shift-field refinement model (thick light bonds) and the deposited model (thin bonds).

For PDB entry 4l9m, the *R* factor after shift-field plus conventional refinement was 40%, compared with 43% for jelly-body plus conventional refinement. A section through the C^α^ traces of the two models is compared with the deposited structure in Fig. 6 ▸. While the lower domain is largely the same in all of the models, the upper domain shows a significant rotation, leading to a displacement of the main chain by up to 4.5 Å. This displacement is largely corrected by shift-field refinement, but jelly-body refinement fails to correct the structure in this case.

**Figure 7:**
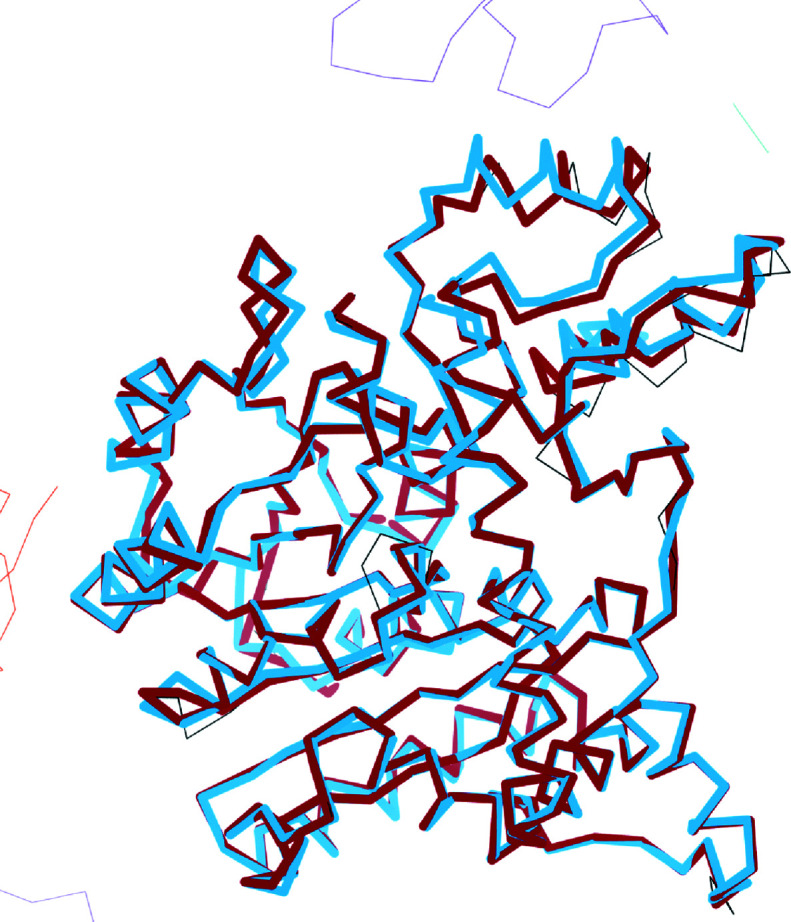
Comparison of models for PDB entry 2d66 showing the C^α^ trace of the jelly-body refined model (thick dark bonds), the shift-field refinement model (thick light bonds) and the deposited model (thin bonds). Some loops have been removed for clarity. Symmetry contacts of the deposited model are also shown (thin light bonds).

By contrast, for PDB entry 2d66 the *R* factor after shift-field plus conventional refinement was 37%, compared with 32% for jelly-body plus conventional refinement. The C^α^ traces of the two models are compared with the deposited structure in Fig. 7 ▸. The centre of the helix at the top of the molecule is displaced by about 3 Å : this is largely corrected by jelly-body refinement but not by shift-field refinement. A likely factor in this behaviour is the presence of part of a neighbouring symmetry molecule close to the surface helix: this portion of the neighbouring molecule does not need to move. Shift-field refinement looks for coordinated motions over large regions and so cannot reconcile the change in behaviour across a molecular boundary, whereas jelly-body refinement can make this distinction through the explicit use of stereochemical restraints. It is likely that the model-morphing technique of Terwilliger *et al.* (2013 ▸) would also work in this case.

## 4. Discussion   

We have shown that shift-field refinement can complement conventional model-refinement methods in two major ways: firstly, the calculation can be conducted at low resolution, with benefits in terms of both speed and radius of convergence; secondly, the calculation is very fast, usually converging in fewer cycles than conventional or jelly-body refinement. The improvement in speed arises largely from the application of coarse-grained shifts to correct large-scale errors in the model, which largely preserves local geometry, thus avoiding the need for stereochemical restraints which can complicate the topology of the refinement target function, and in part from the refinement being performed at low resolution with fewer data.

The lack of stereochemical restraints in the shift-field method does allow gradual distortion of the model geometry to occur as the calculation progresses, so the shift-field refinement calculation must be complemented by conventional refinement in a program such as *REFMAC*5, offsetting some of the speed benefit. Nevertheless, the combined procedure still has a larger radius of convergence than conventional refinement alone and is significantly faster than the jelly-body method, which is often used to increase the radius of convergence (Murshudov *et al.*, 2011 ▸). The method also appears to provide an increased radius of convergence compared with jelly-body refinement but performs less well than jelly-body refinement when different shifts are required across the boundaries between closely neighbouring molecules.

Future work will investigate whether alternating cycles of shift-field refinement and regularization could allow the full speed of shift-field refinement to be exploited without the overhead of a conventional refinement step. Since refinement is a useful tool in the evaluation of molecular replacement or *ab initio* modelling solutions, this may offer benefits for large-scale screening calculations in which a large portion of the Protein Data Bank is searched to try and explain the observed diffraction pattern (Rodríguez *et al.*, 2012 ▸; Simpkin *et al.*, 2018 ▸). A second aim will be to apply the shift-field approach to the refinement of one map against another or against a set of diffraction observations, which has applications both in the use of noncrystallographic symmetry and in the use of cryo-electron microscopy reconstructions to explain X-ray or other diffraction data.

### 4.1. Data and methods   

The computer code and data sets used in this paper are available at https://doi.org/10.15124/5d8e7307-7bde-4e47-875d-5f15f30177bd. The methods described here are also distributed with version 7.1 of the *CCP*4 software suite in the *sheetbend* software.

## Supplementary Material

Computer code and data sets used.: https://doi.org/10.15124/5d8e7307-7bde-4e47-875d-5f15f30177bd

Description of data sets used along with additional metrics and figures. DOI: 10.1107/S2059798320013170/di5041sup1.pdf

## Figures and Tables

**Table 1:** Comparison of geometric validation measures for models refined by the conventional, jelly-body and shift-field refinement protocols The indicators are the percentage of Ramachandran outliers, the percentage of rotamer outliers, the clashscore, the r.m.s.d. of bond lengths from ideal values and the deviation of main-chain *B* values from neighbouring atoms.

	Conventional	Jelly body	Shift field
Ramachandran outliers (%)	5.97	5.24	5.51
Rotamer outliers (%)	13.73	13.84	13.45
Clashscore	19.70	18.60	18.96
Bond r.m.s.d. (Å)	0.0090	0.0090	0.0093
Main-chain *B* standard deviation (Å^2^)	3.05	2.97	3.02
