# Supplementary material for: Shift-field refinement of macromolecular atomic models
Source: Acta Crystallogr D Struct Biol. 2020 Nov 19;76(Pt 12):1192–200. doi: 10.1107/S2059798320013170 (PMC7709196; doi:10.1107/S2059798320013170)
Supplement: Supplementary file 1 [file d-76-01192-sup1.pdf]

STRUCTURAL BIOLOGY

Volume 76 (2020)

Supporting information for article:

Shift-field refinement of macromolecular atomic models

K. Cowtan, S. Metcalfe and P. Bond

Target structure	Target resolution	Search structure	Sequence identity	GESAMT RMSD	PHASER LLG	Initial R-free	Refined R-free	Phase error CR	Phase error JB	Phase error SF
5ZRQ	1.12	1JFR	0.684	0.541	4023.0	0.462	0.356	28.4	28.0	28.3
1UKU	1.45	1VHF	0.470	0.580	569.9	0.496	0.403	32.5	32.0	32.2
4F2E	1.45	4F2F	0.389	0.616	519.8	0.510	0.430	38.7	38.8	38.7
3U04	1.70	4AZ4	0.415	0.696	842.0	0.479	0.395	38.5	38.4	37.3
3ZVD	2.25	1L1N	0.663	0.702	1171.8	0.452	0.372	33.0	32.4	32.8
4YFO	3.35	3DU1	0.556	0.730	892.4	0.482	0.441	71.0	71.2	70.8
5WGI	1.05	5G0G	0.469	0.745	2145.1	0.483	0.329	27.8	27.5	27.3
4MU3	1.12	6FWH	0.459	0.750	894.3	0.518	0.393	32.2	31.1	31.4
5HZH	2.60	5C2K	0.586	0.788	364.5	0.466	0.449	54.6	54.6	54.5
5JEY	2.80	1LQW	0.506	0.789	588.3	0.432	0.409	45.4	45.2	44.8
5WMG	1.15	5O3F	0.430	0.806	575.1	0.517	0.436	38.2	37.9	38.5
4Z79	1.54	1IO0	0.494	0.811	696.7	0.532	0.414	34.0	31.9	32.8
5H9R	1.58	5DUV	0.368	0.841	755.5	0.517	0.419	34.3	30.3	34.1
5KWN	1.42	5IGQ	0.538	0.851	556.7	0.547	0.463	30.2	29.4	29.8
6GKA	1.76	1SQ3	0.224	0.867	385.7	0.492	0.416	41.9	42.1	42.1
1G9K	1.97	3VI1	0.667	0.868	1672.0	0.423	0.350	32.3	32.1	31.7
5M6F	2.39	1Q4Q	0.435	0.877	552.8	0.464	0.434	44.7	43.5	44.0
5ZYS	1.78	3RL7	0.378	0.885	169.4	0.510	0.466	43.8	43.8	43.2
6FM6	1.05	4X68	0.561	0.885	2208.6	0.495	0.363	31.3	29.7	29.2
2NUK	1.20	1RIE	0.472	0.894	150.4	0.527	0.454	46.7	44.0	46.1
5I67	2.60	2ZCI	0.646	0.899	1545.8	0.439	0.383	32.2	30.3	30.7
6BCE	1.75	5ESP	0.467	0.902	711.3	0.508	0.453	47.2	45.3	45.9
3S2N	1.40	1ITQ	0.464	0.907	1849.1	0.512	0.398	36.8	36.4	36.7
4B29	1.72	5JSO	0.441	0.917	1426.4	0.475	0.356	33.6	33.2	33.2
3PKB	1.25	4U6W	0.500	0.937	1397.8	0.493	0.368	32.9	32.3	32.6
3MOK	1.55	3DDR	0.516	0.945	201.4	0.535	0.432	37.9	37.5	37.2
5JEI	1.23	5IKB	0.570	0.947	757.2	0.512	0.390	29.9	29.0	29.9
4PO1	3.40	1XMS	0.628	0.954	951.7	0.365	0.299	32.0	32.2	31.7
5J4K	1.35	2Z43	0.581	0.955	1006.3	0.476	0.378	31.0	29.2	30.1
1FYS	2.00	3WR2	0.419	0.956	243.7	0.499	0.464	44.5	43.5	43.5
5XG4	3.00	1RTF	0.463	0.963	360.8	0.412	0.366	43.4	43.5	42.9
5KTO	1.44	5LQS	0.537	0.966	718.0	0.500	0.411	37.5	36.8	37.4
5JVE	1.10	2J9B	0.472	0.967	310.6	0.548	0.438	35.8	33.0	33.9
1EYB	1.90	4AQ2	0.514	0.973	1573.9	0.482	0.370	34.2	33.4	33.8
5G3Y	1.18	3BE4	0.500	0.975	394.8	0.522	0.448	37.9	35.0	36.0
3K4J	2.00	4MIF	0.482	0.985	1672.4	0.454	0.392	39.0	37.8	38.4
4BAT	1.30	3QYJ	0.461	0.995	1378.3	0.499	0.357	28.4	27.9	28.0
5DUT	1.50	3UYW	0.458	1.006	862.2	0.494	0.365	36.8	36.3	36.5
6FF1	1.30	3I9Z	0.250	1.010	143.9	0.518	0.464	40.0	38.6	38.6
6O5W	1.41	5YVX	0.405	1.029	215.0	0.531	0.479	46.7	46.3	46.1
4B6P	2.30	6CV6	0.415	1.030	344.8	0.456	0.384	32.6	33.0	32.6
5K5M	2.01	4MTP	0.670	1.033	2528.5	0.465	0.368	32.4	32.0	31.8
5EMI	1.12	1JWQ	0.413	1.043	471.5	0.521	0.422	33.1	32.4	32.6
3S4E	1.26	2NT2	0.350	1.045	123.0	0.541	0.502	40.9	33.5	37.9
6D9A	2.90	3GF4	0.443	1.048	559.5	0.490	0.455	43.7	37.8	38.9
4N6K	1.20	4PFB	0.412	1.049	250.8	0.519	0.468	37.9	35.6	35.7
2BBE	1.97	4ZOS	0.156	1.050	296.5	0.542	0.446	43.5	43.3	44.2
3CI1	1.81	3KE5	0.472	1.061	468.2	0.467	0.400	39.7	39.2	39.6
3PFC	1.75	2WTN	0.314	1.063	358.8	0.513	0.437	37.5	35.2	37.0
4WZU	1.88	4NHD	0.404	1.065	740.6	0.490	0.421	37.8	36.4	37.4
3US5	1.38	6HIP	0.402	1.068	389.2	0.499	0.427	41.5	42.3	40.9
4YVO	1.45	3KD7	0.240	1.075	193.6	0.556	0.450	40.8	40.4	40.5
4ZBG	1.25	4RS2	0.393	1.084	227.4	0.532	0.465	41.6	36.9	38.7
1XC8	1.95	1R2Y	0.423	1.096	532.1	0.520	0.465	42.9	41.1	41.8
3A9B	1.20	1BVW	0.452	1.100	534.5	0.517	0.422	40.7	37.1	41.1
2NSM	1.92	3MN8	0.435	1.120	1522.4	0.485	0.392	37.0	35.7	35.9
6GQ4	1.65	3OE3	0.182	1.123	98.2	0.543	0.512	60.1	59.2	60.1
4IQW	2.60	4YCX	0.466	1.130	458.8	0.503	0.467	47.4	40.2	42.1
4YKC	2.70	6FDD	0.233	1.131	130.7	0.513	0.470	50.6	49.1	50.5
5ILS	1.40	5E8G	0.549	1.151	191.2	0.529	0.466	39.0	36.9	36.0
6ESC	2.70	2GUM	0.575	1.151	1200.1	0.447	0.389	35.1	30.6	34.8
5VH1	2.30	2V2U	0.509	1.157	156.7	0.518	0.487	52.6	40.7	44.9
6MGB	1.60	6MGC	0.453	1.159	711.5	0.483	0.415	37.9	37.4	37.2
2R7K	2.10	2R87	0.506	1.163	556.0	0.527	0.458	38.4	37.1	37.0
4NVB	1.17	5AMM	0.406	1.165	645.5	0.532	0.439	38.5	37.2	37.8
5EUS	1.83	5EZB	0.588	1.167	224.7	0.530	0.481	32.1	31.6	31.7
2D1R	1.60	4G36	0.679	1.168	1520.3	0.511	0.424	34.8	28.6	29.4
4HDT	1.60	3BPT	0.377	1.172	337.3	0.493	0.439	39.0	38.0	39.1
5A9A	1.82	5A8S	0.361	1.174	171.8	0.531	0.519	51.1	43.0	46.4
5I2Q	1.94	1AVS	0.295	1.180	95.5	0.548	0.510	54.9	52.2	53.1
4XQM	1.63	3AIH	0.235	1.182	201.4	0.524	0.501	50.6	50.0	49.8
4TYJ	2.45	3D7U	0.418	1.189	421.7	0.505	0.454	40.8	38.0	39.2
5MDU	1.02	5O1Y	0.468	1.206	491.4	0.508	0.415	43.3	40.5	30.5
4W8B	1.15	5OYC	0.400	1.209	479.4	0.517	0.434	43.7	41.6	43.0
4J7T	3.20	2Q7M	0.363	1.213	288.6	0.453	0.415	40.8	40.3	40.6
5NOA	1.26	4CE7	0.328	1.214	215.1	0.530	0.490	43.9	37.1	38.2
5BVR	1.46	1WKU	0.502	1.215	247.3	0.522	0.466	34.3	31.8	32.5
2BHZ	1.20	3VGG	0.369	1.221	651.8	0.530	0.462	43.7	40.1	40.6
5C1C	1.80	1XG2	0.341	1.228	908.7	0.476	0.426	43.0	42.6	42.7
3ESS	1.19	1XK9	0.435	1.229	353.9	0.539	0.469	43.8	42.1	41.8
2CG3	2.60	4AXH	0.451	1.242	957.2	0.496	0.477	36.8	32.5	33.9
4WPG	1.10	1VL0	0.410	1.242	351.0	0.510	0.436	40.9	37.5	36.7
4XHV	1.23	4UU6	0.217	1.244	63.6	0.560	0.543	59.6	58.0	58.7
3FD3	1.70	3ISP	0.424	1.265	284.8	0.534	0.468	40.4	39.8	37.0
4LQT	1.10	3VIC	0.233	1.266	144.8	0.532	0.535	84.0	79.8	40.3
2H30	1.60	4HQS	0.295	1.267	162.3	0.545	0.455	38.6	37.2	38.0
3E8X	2.09	3DQP	0.352	1.270	541.6	0.500	0.428	39.0	37.6	37.1
5O1Z	3.40	5MDU	0.468	1.281	239.7	0.434	0.422	42.7	40.2	39.6
4CST	1.10	5LNG	0.430	1.283	188.2	0.540	0.490	37.0	33.8	34.8

Target structure	Target resolution	Search structure	Sequence identity	GESAMT RMSD	PHASER LLG	Initial R-free	Refined R-free	Phase error CR	JB	SF
5I9J	1.74	3P0L	0.369	1.296	213.5	0.544	0.529	74.9	73.9	62.2
6EVG	1.10	2YXW	0.412	1.299	446.6	0.517	0.443	39.9	31.2	40.3
4NOK	2.50	5H0I	0.425	1.301	615.3	0.500	0.480	44.5	43.8	43.4
3SAU	1.65	4PDG	0.414	1.304	114.1	0.548	0.529	42.4	33.5	39.4
5ZHJ	1.75	1P9P	0.435	1.305	139.5	0.525	0.516	47.2	35.1	35.4
5CLA	1.54	2B6C	0.365	1.307	136.2	0.524	0.491	53.5	50.4	50.9
5ACK	1.24	4C0T	0.513	1.309	355.0	0.523	0.427	37.2	33.8	36.2
5UU2	1.22	2WFU	0.304	1.310	247.5	0.514	0.483	53.3	53.1	53.1
4D74	1.57	1LJU	0.250	1.319	82.3	0.548	0.496	51.0	50.5	50.2
3Q71	2.20	5LAU	0.215	1.327	155.6	0.532	0.498	48.4	45.7	45.3
5EXJ	1.62	4U0O	0.469	1.329	216.2	0.541	0.530	52.7	46.3	47.1
5K1Q	1.55	3GKA	0.426	1.329	733.5	0.484	0.416	40.5	37.7	38.8
3OJG	1.60	4KF1	0.350	1.335	273.6	0.538	0.496	46.9	44.4	44.7
6DN4	1.99	1L9Y	0.336	1.335	165.9	0.544	0.498	49.0	40.7	42.4
6INC	1.60	1XV2	0.326	1.338	168.5	0.543	0.503	43.0	40.9	41.3
3FF2	1.90	4KVH	0.170	1.347	76.4	0.556	0.552	60.9	57.6	57.6
6KBX	1.22	2BDV	0.369	1.355	137.3	0.513	0.510	52.1	49.4	49.1
4ZRG	2.70	1G4M	0.585	1.356	280.9	0.529	0.492	50.0	40.6	41.0
5W0F	1.60	5FFV	0.274	1.358	44.0	0.535	0.482	51.8	51.5	50.3
2YLZ	2.00	3UOZ	0.428	1.361	556.5	0.523	0.504	56.4	35.4	41.9
2XVZ	2.40	2XWP	0.361	1.366	296.2	0.496	0.462	44.3	39.4	39.1
3WH2	1.30	6A7T	0.294	1.378	117.7	0.523	0.466	52.4	52.0	48.6
4MZB	1.04	2HZF	0.283	1.378	53.5	0.575	0.579	53.6	45.5	34.5
5H8T	1.21	1LIC	0.364	1.378	67.5	0.538	0.534	61.8	42.2	44.1
3PLB	1.18	1GVZ	0.354	1.384	91.7	0.548	0.526	54.8	46.5	52.9
3HS4	1.09	4TWM	0.309	1.393	172.8	0.544	0.482	51.7	48.4	46.0
5BOW	1.31	3F62	0.144	1.397	123.8	0.566	0.531	53.2	50.3	50.6
6OPI	3.00	2I6L	0.477	1.397	192.1	0.535	0.528	59.5	54.4	55.5
4NBR	1.35	4BO2	0.353	1.418	176.4	0.539	0.489	50.6	48.1	48.9
4BDK	3.30	3ZXT	0.371	1.419	210.1	0.471	0.445	48.3	45.1	44.8
3TG2	1.10	3HB7	0.223	1.429	180.7	0.559	0.489	42.1	39.4	42.2
3H3E	2.75	2P4Z	0.344	1.430	255.5	0.492	0.444	49.4	47.7	47.7
4PJN	2.00	1KK8	0.379	1.430	572.3	0.510	0.477	48.7	43.8	43.8
5UL6	1.45	2OAW	0.333	1.430	35.0	0.530	0.538	73.5	60.5	67.1
5YFX	2.70	2YRF	0.329	1.434	235.7	0.538	0.501	47.8	45.8	45.9
2HYK	1.30	5DXD	0.378	1.441	131.3	0.523	0.510	62.0	55.8	52.2
4AV8	3.35	5LTD	0.235	1.443	261.2	0.527	0.467	45.0	42.2	42.1
4N9S	1.06	4R8X	0.271	1.446	128.3	0.545	0.533	48.8	38.5	38.4
4MXD	1.45	2XMZ	0.289	1.447	116.2	0.548	0.520	48.3	40.2	47.6
6NYW	2.19	6B5D	0.426	1.449	242.0	0.509	0.456	43.2	37.0	38.4
6E1A	3.09	3RE2	0.539	1.452	594.3	0.480	0.446	42.2	39.8	38.9
5JNT	1.36	1LJL	0.220	1.453	72.5	0.537	0.520	56.4	51.6	51.5
5VIX	1.55	5Y3T	0.221	1.458	22.4	0.567	0.560	84.4	84.4	82.0
4EX9	3.15	4GIJ	0.439	1.460	410.1	0.464	0.398	34.9	33.2	34.4
5ZUN	1.35	6EIC	0.350	1.466	191.6	0.543	0.494	45.2	39.9	40.1
4X8G	3.29	5HP5	0.589	1.468	556.8	0.455	0.398	37.7	32.8	33.5
3CLM	1.14	4E0C	0.244	1.469	88.5	0.534	0.510	57.8	52.2	53.9
4MYQ	1.90	3QPO	0.256	1.469	157.0	0.508	0.486	49.4	46.6	46.6
4OU4	2.40	1JJI	0.316	1.470	238.1	0.527	0.484	51.0	47.8	45.4
6A9V	2.90	3IG4	0.271	1.470	270.5	0.504	0.469	46.1	40.7	42.3
5LW3	1.19	3ZPP	0.218	1.476	295.0	0.549	0.534	81.7	78.0	55.9
3V2B	2.20	2DX6	0.276	1.477	55.3	0.564	0.543	63.1	60.4	52.1
5KPZ	2.40	4NB4	0.239	1.477	167.4	0.536	0.501	51.2	49.7	49.8
1HV7	1.70	1GVZ	0.335	1.478	98.8	0.542	0.529	65.8	62.8	56.3
1X55	1.80	1WYD	0.355	1.480	287.2	0.540	0.517	52.0	41.5	48.2
5D9L	2.15	3A62	0.548	1.480	165.3	0.486	0.456	52.7	47.1	47.0
5DXX	1.45	3KRZ	0.292	1.480	140.9	0.537	0.520	53.7	49.1	49.8
4JED	1.10	3M0F	0.233	1.481	70.8	0.567	0.566	76.3	59.4	49.0
5ARB	1.15	4M1Z	0.323	1.481	180.4	0.539	0.489	49.2	45.2	39.2
2X5Y	1.01	5NGO	0.136	1.482	93.8	0.550	0.511	58.0	54.7	51.9
3LA9	2.05	3NTN	0.427	1.486	98.8	0.542	0.554	74.7	72.8	72.6
6CN3	3.35	3X08	0.330	1.486	380.3	0.484	0.445	38.8	35.3	36.1
6FZG	1.79	3IPQ	0.269	1.486	130.0	0.530	0.491	48.3	45.8	47.6
3PL3	1.18	5W0A	0.474	1.493	505.6	0.528	0.455	41.7	40.4	41.4
5IR2	2.08	5X15	0.204	1.496	180.2	0.535	0.515	47.9	46.6	45.2
5ZNC	1.66	3UAZ	0.239	1.496	147.2	0.534	0.506	48.8	46.8	47.2
5IT6	1.55	4LBQ	0.238	1.500	195.2	0.517	0.493	43.8	40.2	40.2
3DBC	3.35	5D9K	0.247	1.502	116.2	0.512	0.475	47.0	44.7	46.5
5J93	1.10	5V5K	0.278	1.502	48.3	0.571	0.572	87.1	87.1	86.7
6GFA	2.00	6EOB	0.393	1.502	228.3	0.536	0.500	44.7	37.5	38.4
5LAQ	2.40	3HQW	0.253	1.504	166.3	0.516	0.487	50.4	43.5	45.4
6GTO	2.97	4B0F	0.077	1.507	41.2	0.619	0.647	77.0	76.3	77.9
4NUX	2.29	3VBC	0.294	1.508	46.4	0.517	0.524	70.6	67.3	70.7
4JG2	1.30	5ODL	0.250	1.513	59.3	0.534	0.519	50.0	42.6	46.0
2D66	2.18	5AX8	0.384	1.515	400.0	0.505	0.458	42.7	33.2	41.7
5HRV	1.70	6G8R	0.176	1.516	57.7	0.536	0.516	53.7	53.2	53.8
5L4S	1.41	1SNY	0.285	1.518	186.7	0.565	0.529	59.0	58.1	55.6
3UBB	2.60	2NR9	0.376	1.520	374.0	0.455	0.412	37.7	37.4	37.8
3BW4	2.00	4IQL	0.330	1.521	186.5	0.514	0.493	45.0	43.3	43.5
5Y8B	2.40	5Y8A	0.457	1.521	90.7	0.534	0.529	53.9	36.3	38.4
6EQ2	1.80	3FK9	0.254	1.521	86.5	0.551	0.514	48.1	45.5	46.1
4ALQ	1.48	4OY7	0.218	1.527	70.0	0.538	0.542	63.0	49.6	60.8
4UTL	1.23	3L5A	0.274	1.529	83.0	0.529	0.514	51.2	42.3	47.0
5PHS	2.54	6DQB	0.393	1.531	194.8	0.519	0.523	59.1	53.8	56.6
5MQO	2.50	4QK0	0.359	1.532	408.2	0.495	0.478	53.5	41.2	44.0
5TDB	1.10	3NIJ	0.477	1.534	72.5	0.539	0.458	50.1	49.5	50.2
5EXN	1.49	2OLG	0.327	1.540	356.6	0.525	0.459	42.0	41.6	41.8
4RTR	2.39	1YFL	0.257	1.544	76.1	0.535	0.538	64.5	64.5	59.7
1Z2M	2.50	6MDH	0.505	1.545	24.0	0.597	0.593	82.1	82.4	82.1

Target structure	Target resolution	Search structure	Sequence identity	GESAMT RMSD	PHASER LLG	Initial R-free	Refined R-free	Phase error		
								CR	JB	SF
3JS5	1.94	1JF8	0.214	1.546	40.4	0.512	0.515	72.6	72.4	58.7
5OPF	1.08	4OY7	0.299	1.546	118.6	0.544	0.483	46.7	45.9	46.2
3LHI	1.33	1VL1	0.243	1.547	74.2	0.543	0.542	79.5	75.7	47.2
2A0M	1.60	3M1R	0.231	1.549	91.1	0.525	0.505	54.4	46.2	45.9
3G28	1.64	4COC	0.208	1.551	53.7	0.528	0.533	65.8	60.8	60.4
5I4N	1.54	3QGY	0.285	1.552	156.9	0.538	0.509	43.3	40.4	42.7
5G28	1.57	4HYJ	0.197	1.553	159.6	0.522	0.479	55.9	52.4	51.1
6BPW	2.43	4WVZ	0.188	1.553	123.0	0.499	0.464	55.9	53.5	51.6
3ET9	2.80	5DHX	0.493	1.557	94.9	0.510	0.506	62.7	62.1	56.8
4X1G	2.25	1K4W	0.273	1.560	86.6	0.564	0.554	56.3	48.0	50.7
3A2I	3.27	1N4H	0.251	1.567	91.0	0.547	0.518	62.8	64.0	57.1
4F8X	1.47	4BS0	0.305	1.567	99.6	0.542	0.532	72.6	51.6	51.2
5N13	1.16	5JI8	0.235	1.568	108.2	0.560	0.506	51.3	50.7	42.3
4YUO	1.20	2OSE	0.263	1.573	96.8	0.544	0.504	46.2	41.2	40.6
4LU3	2.00	4TWL	0.285	1.575	358.5	0.505	0.466	48.5	44.3	48.4
5AUL	1.10	1CJ1	0.323	1.575	83.0	0.545	0.501	50.0	49.4	49.2
6MGL	1.50	6P2L	0.383	1.575	169.5	0.545	0.545	81.4	80.5	52.7
4H4B	2.90	4OHF	0.337	1.576	209.2	0.519	0.499	52.9	46.6	47.7
5MAF	2.80	6GT1	0.286	1.579	56.3	0.552	0.534	85.2	84.9	85.2
5VML	1.70	3JQ6	0.251	1.579	153.6	0.566	0.541	53.4	51.9	49.4
5OY2	1.36	5FUK	0.344	1.580	56.9	0.535	0.533	77.2	63.0	63.5
5ZLJ	1.96	5K0D	0.276	1.581	217.3	0.547	0.534	57.1	49.7	49.8
3O5R	1.10	5U9A	0.231	1.583	115.8	0.546	0.502	52.9	51.5	51.7
4LHL	1.43	6HOS	0.302	1.583	104.6	0.548	0.542	64.2	51.8	52.2
5HJK	2.00	2ZZN	0.353	1.583	97.4	0.547	0.594	87.0	88.3	87.3
3P7Y	1.20	3L5A	0.249	1.587	46.0	0.552	0.549	82.9	82.1	76.7
5C7H	1.30	3V0T	0.283	1.588	139.6	0.551	0.519	47.6	39.6	42.0
6D4T	1.54	2QR6	0.243	1.588	151.8	0.507	0.475	45.7	43.7	42.3
3W8F	1.45	2B4Q	0.324	1.590	56.8	0.548	0.536	55.2	52.2	54.2
6DZ0	1.62	3SCZ	0.244	1.591	90.1	0.555	0.548	53.1	47.7	49.0
2ZUI	1.50	5KYO	0.381	1.592	135.1	0.571	0.561	67.6	44.5	45.1
5EP7	1.20	5DKH	0.206	1.593	78.5	0.524	0.487	54.6	51.9	48.0
5TG0	1.44	3H8U	0.158	1.593	54.3	0.539	0.544	71.0	68.2	66.9
4YC0	1.50	6DRE	0.284	1.594	51.9	0.544	0.533	75.9	73.3	58.0
6BT1	1.48	3G6S	0.214	1.596	92.1	0.546	0.524	52.8	43.1	47.5
1UP0	1.75	6IDW	0.313	1.597	261.4	0.542	0.529	49.6	43.6	45.6
4GK6	1.65	4AOZ	0.323	1.599	53.9	0.534	0.535	64.3	62.5	47.2
5T9C	1.48	1O1Z	0.262	1.600	110.8	0.526	0.499	49.3	45.9	47.9
4MX6	1.10	4NQ8	0.233	1.605	35.3	0.543	0.543	88.9	88.9	85.4
5NY7	1.31	5G3O	0.191	1.609	129.7	0.555	0.518	50.5	44.3	44.5
5JIX	1.47	4KU8	0.478	1.614	98.7	0.528	0.502	55.3	53.8	51.7
6B7I	1.54	3SCZ	0.352	1.616	102.0	0.522	0.487	50.2	43.4	43.2
2RBK	1.00	3R4C	0.385	1.617	233.3	0.535	0.457	38.1	35.4	36.9
4U5X	1.90	1Z0D	0.255	1.618	47.9	0.547	0.562	62.4	61.9	60.0
1H2N	2.84	6AVS	0.288	1.619	142.4	0.554	0.543	62.3	61.4	60.4
6B2P	3.01	3QGY	0.230	1.621	80.4	0.495	0.485	60.3	45.8	50.5
4OPC	1.40	3OZ2	0.243	1.625	158.6	0.537	0.510	51.4	47.9	47.1
5GZC	1.08	1ULG	0.171	1.631	86.9	0.548	0.500	45.6	43.4	45.8
5WNK	3.11	4M69	0.336	1.638	308.9	0.504	0.466	42.5	37.9	38.9
5IMA	1.04	3ZVJ	0.239	1.639	26.8	0.532	0.537	83.1	82.3	65.2
4N4K	2.20	1FGJ	0.316	1.640	135.5	0.535	0.524	68.3	60.6	58.5
4WJE	1.29	3S6D	0.261	1.642	78.6	0.559	0.550	65.5	44.1	45.5
5ZH9	1.06	3WP6	0.309	1.644	107.7	0.543	0.502	53.0	50.5	49.9
1OD8	1.05	4PMU	0.361	1.645	119.5	0.550	0.531	53.0	44.5	40.1
6C2Q	2.17	3X43	0.301	1.645	251.9	0.516	0.494	50.8	47.0	45.8
4AYS	3.15	5GTW	0.285	1.646	164.0	0.567	0.557	62.2	47.8	53.6
5JKN	3.00	6K6L	0.283	1.647	388.9	0.474	0.432	49.1	39.2	41.1
6GAE	1.80	6RF7	0.239	1.648	157.0	0.492	0.459	46.9	44.2	43.9
6AYD	3.00	5UOX	0.277	1.649	128.2	0.475	0.474	62.8	53.9	53.4
5WJD	2.00	3EFA	0.210	1.650	27.1	0.593	0.573	83.4	83.2	81.0
4ZA8	1.06	4IWS	0.397	1.651	221.5	0.540	0.518	49.6	39.9	38.2
3NVS	1.02	3SLH	0.302	1.653	97.7	0.524	0.509	54.5	48.4	47.9
4DE7	2.99	2BO8	0.234	1.653	74.6	0.532	0.509	63.4	56.3	58.9
5ETB	1.29	6MR4	0.208	1.657	67.9	0.525	0.473	48.9	46.4	47.9
4YU1	1.02	3V0T	0.248	1.660	66.0	0.542	0.537	80.3	57.0	77.3
5VDC	1.60	5U2J	0.519	1.660	56.4	0.560	0.536	51.8	49.9	50.6
2YD6	1.35	3PXJ	0.511	1.662	95.4	0.554	0.525	51.8	48.3	40.3
4PMS	2.80	4FC0	0.304	1.663	90.2	0.512	0.500	58.4	51.1	51.5
4UYT	1.03	5FV6	0.331	1.663	79.6	0.537	0.530	47.2	37.3	39.2
4WJM	1.70	1VM7	0.233	1.666	90.2	0.552	0.541	76.4	66.0	50.5
3S5Q	1.85	3U1X	0.253	1.671	64.1	0.505	0.508	77.7	76.6	75.4
4GVZ	2.96	6FH2	0.273	1.672	73.2	0.479	0.488	70.5	66.9	70.2
5IKD	1.11	5C2I	0.280	1.674	223.6	0.523	0.476	47.5	45.4	40.6
4OY3	1.20	3BSF	0.243	1.675	51.0	0.536	0.521	50.9	43.4	46.1
2PQC	1.60	1X8R	0.282	1.680	88.6	0.509	0.508	79.4	79.5	67.5
6AUB	1.65	3P86	0.302	1.680	97.0	0.548	0.545	74.0	53.2	45.9
2GG2	1.00	2V6C	0.222	1.684	41.5	0.556	0.550	84.3	82.9	71.9
2IVJ	1.46	6JYV	0.309	1.684	74.8	0.562	0.556	62.4	54.1	53.6
3ORU	1.11	3DI4	0.282	1.684	61.4	0.543	0.531	83.3	83.2	69.1
4YPZ	1.71	3QUV	0.426	1.684	67.8	0.560	0.542	58.9	37.7	38.1
4LUU	1.95	4OKZ	0.221	1.686	46.0	0.534	0.532	81.3	81.9	82.2
4PWP	1.80	4GXZ	0.215	1.686	39.7	0.548	0.535	75.8	75.5	76.0
4R0E	3.00	4NZ0	0.317	1.686	255.7	0.507	0.481	53.4	38.9	47.9
4HIK	1.64	4OWX	0.193	1.695	29.1	0.555	0.576	83.6	84.1	83.0
5IH1	1.31	5IGT	0.377	1.695	95.9	0.535	0.523	71.1	38.8	39.5
1X3M	2.20	4H0O	0.307	1.704	213.5	0.492	0.460	44.8	38.3	43.6
6H5G	1.04	5SWU	0.241	1.705	74.0	0.566	0.525	50.8	47.3	45.2
2OVJ	1.49	2XS6	0.272	1.707	101.4	0.543	0.522	60.8	59.4	57.0
4PAK	1.20	4O94	0.307	1.707	101.1	0.537	0.535	86.5	86.4	42.6

Target structure	Target resolution	Search structure	Sequence identity	GESAMT RMSD	PHASER LLG	Initial R-free	Refined R-free	Phase error CR	Phase error JB	Phase error SF
5FM2	3.30	1OL6	0.223	1.707	67.8	0.521	0.527	58.3	52.1	54.0
6BR5	2.04	4GEZ	0.233	1.708	182.2	0.561	0.543	57.3	54.6	53.7
6C93	2.67	2Q9F	0.258	1.709	167.9	0.506	0.500	58.9	45.2	50.4
3QZ6	2.00	4B5V	0.290	1.710	157.1	0.514	0.502	68.2	47.2	48.3
5VD6	1.20	4PV6	0.175	1.710	40.3	0.565	0.566	88.2	88.0	75.8
5N2X	1.21	6F8B	0.328	1.711	55.6	0.545	0.541	88.5	88.2	88.3
4UQX	1.20	1ZBP	0.228	1.712	56.9	0.535	0.533	89.0	88.6	75.8
3CH0	1.50	3NO3	0.242	1.713	64.6	0.554	0.558	75.1	69.4	55.3
6BO0	1.20	4K6X	0.263	1.713	69.1	0.552	0.540	54.7	51.6	49.7
1EX9	2.54	5H6B	0.209	1.714	27.9	0.559	0.542	72.1	71.5	70.2
4OOX	1.03	3D26	0.384	1.716	98.7	0.544	0.537	54.3	50.7	43.5
5GI7	1.20	4FD7	0.245	1.717	37.8	0.551	0.551	85.9	85.9	82.0
4KEF	1.10	3L50	0.165	1.719	70.8	0.529	0.508	54.6	48.1	53.9
4FHV	2.10	5JNB	0.292	1.720	97.9	0.550	0.536	56.4	48.6	49.6
2FI1	1.40	2YBD	0.219	1.723	50.1	0.531	0.537	62.6	57.2	58.2
3WFG	1.40	5UNJ	0.201	1.724	55.2	0.544	0.544	85.2	84.5	56.8
5FZ0	2.42	5YKN	0.424	1.725	551.1	0.517	0.486	50.0	41.1	39.5
4D7R	1.80	4D7Q	0.453	1.726	109.7	0.520	0.513	57.3	46.2	53.3
4K8W	1.67	4ME8	0.247	1.728	65.4	0.546	0.509	52.5	51.4	51.6
2DY5	2.71	4RAJ	0.227	1.729	72.7	0.513	0.502	63.9	43.1	58.5
4B62	1.45	3WPW	0.352	1.729	133.8	0.540	0.516	44.5	42.9	43.0
6HHF	2.90	5M57	0.236	1.730	41.6	0.527	0.519	77.0	77.3	78.4
5JXH	2.00	4LVN	0.193	1.733	85.3	0.539	0.541	72.0	69.8	72.0
5F7V	1.40	5YSE	0.220	1.740	57.2	0.555	0.555	83.6	83.5	78.8
5VZC	1.55	3PMN	0.347	1.740	108.5	0.541	0.528	55.6	52.8	46.0
3PPT	1.28	3EM0	0.222	1.741	36.7	0.560	0.562	75.2	73.8	73.5
4U63	1.67	1IQU	0.356	1.746	203.1	0.529	0.511	51.9	47.9	46.6
4DVC	1.20	4ZL7	0.281	1.751	42.7	0.539	0.530	60.4	56.6	44.8
6HM1	1.54	4DWS	0.262	1.754	50.7	0.539	0.538	82.9	82.7	73.6
2WOY	1.50	4OFQ	0.356	1.755	99.4	0.532	0.530	75.3	66.8	44.0
3NY4	1.22	2WUQ	0.209	1.762	42.0	0.555	0.547	77.9	74.5	61.2
4XT0	2.06	4QQ7	0.267	1.764	81.3	0.544	0.534	52.9	45.0	47.3
5XQK	1.12	3OOY	0.261	1.764	173.2	0.551	0.543	58.7	49.4	47.1
4M36	2.04	4HSG	0.246	1.765	49.2	0.544	0.538	78.3	77.0	73.1
5BQF	1.45	4WEQ	0.597	1.765	652.8	0.523	0.468	45.8	45.8	39.3
4KVK	1.98	6ERC	0.214	1.772	84.6	0.537	0.531	80.1	71.4	63.4
5MTU	1.00	2XD3	0.237	1.772	45.2	0.556	0.556	88.8	88.6	84.6
5VQK	3.10	1XV9	0.237	1.772	44.3	0.555	0.567	70.5	67.9	65.3
4HOR	1.86	6C6K	0.568	1.773	117.3	0.544	0.541	63.4	46.3	48.5
4QX2	2.90	2C9K	0.285	1.773	236.1	0.460	0.455	59.6	48.6	49.7
4D7Y	1.44	4RSU	0.186	1.774	37.5	0.495	0.531	77.8	76.6	70.1
5EW6	2.29	5XTW	0.437	1.776	339.8	0.494	0.459	48.4	46.8	43.1
3KWK	1.54	3GAG	0.217	1.777	50.3	0.547	0.534	69.2	66.0	58.2
5XCY	1.20	1UP3	0.301	1.779	84.0	0.545	0.523	60.0	51.9	51.3
1LRM	2.10	3E2T	0.595	1.781	214.8	0.533	0.509	49.5	42.5	46.9
2OZG	2.00	4MY0	0.276	1.782	110.7	0.523	0.515	65.6	43.9	51.1
6G3F	2.22	6IEM	0.314	1.785	244.0	0.557	0.540	55.5	48.0	50.6
4XPL	1.95	2AE6	0.164	1.788	30.0	0.538	0.542	72.5	70.0	59.9
5T79	1.86	4PMJ	0.277	1.790	75.1	0.549	0.538	64.4	51.1	62.9
3V4B	1.40	3TJ4	0.247	1.791	68.8	0.553	0.551	84.9	84.8	49.5
4X2F	1.49	1OGU	0.230	1.797	67.2	0.539	0.540	77.1	73.4	73.9
3NWO	1.90	4X00	0.172	1.804	27.2	0.535	0.523	84.9	84.6	80.7
5FAI	1.80	3O7B	0.310	1.805	180.1	0.559	0.520	47.1	42.5	43.4
4ZJU	1.20	4IS3	0.199	1.807	56.4	0.550	0.547	85.9	85.6	53.0
6OS6	1.33	5JXM	0.234	1.807	67.2	0.546	0.546	82.2	72.1	69.5
4C2Q	2.40	3SCL	0.430	1.808	251.2	0.541	0.523	68.3	33.8	39.2
5WRL	3.10	4IKN	0.271	1.808	95.4	0.546	0.501	49.8	41.1	45.8
6DEP	2.15	1UPA	0.254	1.808	257.0	0.550	0.536	53.5	43.1	44.7
4PVK	1.30	5L6F	0.263	1.809	166.4	0.557	0.543	49.2	45.8	45.1
4QQN	2.08	4M38	0.248	1.811	102.0	0.558	0.547	65.2	59.0	54.3
6OHK	1.20	4OXX	0.212	1.815	49.2	0.551	0.543	82.0	80.8	59.4
2QDX	1.55	1QFJ	0.163	1.817	51.6	0.530	0.525	81.4	79.5	70.5
4YSV	2.77	4ZM3	0.254	1.823	152.5	0.540	0.516	62.3	51.1	49.6
3QO7	2.55	1GGM	0.174	1.824	66.5	0.533	0.521	74.5	69.7	68.4
6QQH	1.38	4MN6	0.165	1.824	29.8	0.558	0.523	83.9	83.3	83.0
1E24	2.35	4UP9	0.408	1.832	336.0	0.548	0.513	49.6	45.3	44.9
1Z35	2.40	1YBF	0.163	1.833	116.3	0.504	0.498	57.9	54.8	61.0
3QXH	1.36	6CVU	0.225	1.835	34.9	0.565	0.556	82.1	81.8	79.7
5ULU	2.85	4ZPM	0.273	1.837	105.5	0.537	0.529	55.6	47.9	50.1
4L2H	1.46	4NA0	0.290	1.839	289.1	0.525	0.486	52.2	48.5	47.7
4YZJ	2.11	6OS5	0.252	1.839	51.5	0.526	0.528	75.5	72.7	72.0
5XJH	1.54	5G5M	0.153	1.841	35.9	0.555	0.557	83.4	83.4	83.5
3RD5	1.50	2QQ5	0.219	1.844	71.4	0.543	0.539	73.1	68.7	62.0
5GUV	3.08	4FT2	0.340	1.844	150.5	0.506	0.498	67.0	63.4	62.0
2P7A	2.30	1NQ7	0.202	1.849	47.3	0.543	0.531	83.4	82.2	73.5
5D6L	3.20	5WIU	0.306	1.853	70.3	0.481	0.479	76.7	77.3	73.2
3CU3	2.00	2RGQ	0.156	1.855	41.2	0.544	0.528	83.4	83.5	74.3
4M9H	2.39	4FO6	0.360	1.859	72.1	0.534	0.531	70.8	56.0	61.5
5WCT	2.30	4CGS	0.204	1.862	35.9	0.546	0.541	79.3	78.4	66.4
6G28	1.23	1T5J	0.233	1.869	48.6	0.568	0.568	85.9	85.9	79.2
5IAI	1.60	4RYA	0.280	1.874	85.7	0.543	0.538	83.2	81.4	64.1
5N3G	1.16	4QMN	0.266	1.875	65.1	0.550	0.545	76.7	63.4	75.5
4TNM	2.90	5XJG	0.199	1.879	69.2	0.479	0.478	69.8	67.4	64.4
3MFI	1.76	3H4B	0.229	1.888	136.3	0.550	0.538	70.4	62.8	63.4
4DIA	2.00	4MUP	0.294	1.888	30.5	0.547	0.537	85.8	85.8	83.8
6N6X	3.00	4ZTK	0.172	1.892	133.6	0.560	0.509	57.0	55.0	55.0
1YLH	1.70	2PC9	0.466	1.893	155.6	0.531	0.515	52.5	42.1	47.1
5YBJ	2.34	1NFI	0.259	1.893	55.3	0.538	0.551	89.5	89.5	90.7
1O1Z	1.60	2OTD	0.260	1.894	102.3	0.548	0.557	68.9	64.4	54.3

Target structure	Target resolution	Search structure	Sequence identity	GESAMT RMSD	PHASER LLG	Initial R-free	Refined R-free	Phase error		
								CR	JB	SF
1JHD	1.70	1R6X	0.301	1.900	70.9	0.549	0.554	77.6	75.2	60.6
2A0N	1.64	4WD0	0.204	1.901	55.5	0.570	0.569	87.2	87.5	79.1
6DR1	2.49	4GFU	0.220	1.901	54.3	0.517	0.506	78.2	74.8	71.9
4M8U	1.45	3ZO9	0.339	1.906	66.8	0.534	0.529	82.3	82.0	63.7
5IGH	1.55	5UXC	0.411	1.908	66.9	0.551	0.541	77.4	61.6	44.1
3O0F	1.94	2YB4	0.259	1.911	64.7	0.529	0.529	78.0	71.2	51.5
5HCT	1.36	3WB4	0.265	1.914	99.8	0.542	0.516	57.0	53.6	56.9
2YZA	3.02	2H34	0.203	1.915	44.2	0.537	0.557	71.8	72.1	68.6
4Q8W	1.14	6FV5	0.239	1.917	59.5	0.536	0.528	87.2	87.2	47.9
4FM1	3.00	5MDN	0.341	1.923	178.7	0.514	0.519	66.2	62.9	63.4
5P64	1.07	3WB4	0.265	1.928	97.3	0.542	0.518	56.4	51.2	55.7
1ZWL	2.80	5VEG	0.182	1.935	28.0	0.585	0.569	77.8	77.5	73.6
6NX0	1.54	6E1C	0.302	1.936	78.2	0.544	0.536	73.7	71.7	61.0
3MGW	1.75	6G1I	0.175	1.938	27.2	0.523	0.505	82.5	81.8	77.6
4RCZ	1.39	5IZM	0.243	1.943	47.0	0.553	0.538	80.2	79.2	64.6
5ZTH	3.22	3BT7	0.218	1.947	53.1	0.551	0.533	71.2	70.6	67.4
4JG5	2.34	3LIU	0.237	1.948	102.9	0.540	0.521	64.0	61.4	60.8
5EMU	1.50	3F4W	0.165	1.949	40.0	0.548	0.527	85.0	85.1	85.2
5EPG	3.39	1DGJ	0.277	1.950	84.0	0.530	0.519	72.0	65.7	63.8
3TEP	2.03	3OKZ	0.245	1.954	43.8	0.567	0.547	80.1	80.1	70.5
6Q4U	2.01	1D9F	0.412	1.955	127.1	0.520	0.524	71.1	64.0	71.2
6IQB	1.75	1VJG	0.167	1.956	43.5	0.557	0.568	76.1	63.0	75.4
5F3Y	3.41	3PVL	0.508	1.958	283.2	0.514	0.473	49.4	38.9	40.5
4AAN	1.22	6E1C	0.291	1.959	88.7	0.527	0.518	61.7	44.9	51.2
5H9K	1.35	3EBK	0.151	1.959	37.7	0.529	0.531	68.2	64.7	69.8
6EFY	2.90	6EG1	0.474	1.965	375.1	0.497	0.433	45.5	41.5	43.0
3UJ9	1.24	2O57	0.223	1.968	44.0	0.533	0.529	83.8	83.6	80.4
6E13	2.35	3P2U	0.232	1.971	66.3	0.545	0.528	69.3	68.4	64.7
6HOX	1.95	3RMY	0.239	1.982	83.9	0.526	0.533	66.5	55.4	53.7
6M7U	3.20	4EYI	0.303	1.984	63.9	0.567	0.549	67.9	64.3	59.7
5TGK	1.91	1JI0	0.263	1.986	35.4	0.515	0.513	83.3	83.1	76.4
4AFE	2.59	3TUB	0.226	1.993	67.7	0.518	0.522	74.7	71.9	68.3
4QKL	1.71	5JNB	0.136	1.998	15.2	0.490	0.496	83.6	84.6	82.7
5T8B	1.50	5ZQ3	0.253	1.998	43.9	0.563	0.565	81.7	82.2	72.5
4XKN	1.85	4OEV	0.215	2.002	56.1	0.567	0.564	84.8	83.9	73.7
5CXW	1.75	4OVD	0.181	2.003	83.5	0.565	0.555	77.9	76.2	69.5
5E85	2.57	1DKZ	0.467	2.006	132.2	0.503	0.504	61.4	61.4	59.8
3WC3	1.50	4CJ0	0.221	2.010	54.4	0.514	0.513	83.4	82.8	71.9
3CJM	1.50	2WUQ	0.184	2.025	53.3	0.566	0.559	64.8	57.8	56.2
5JJB	2.31	4EZE	0.279	2.025	39.6	0.545	0.541	77.1	75.8	70.6
6DS0	3.19	4EA3	0.276	2.029	96.2	0.549	0.534	64.0	53.5	58.4
2XE1	2.50	6EHE	0.172	2.030	134.0	0.564	0.564	84.3	84.3	83.9
5PER	2.00	6BYA	0.194	2.034	39.5	0.571	0.534	62.5	61.2	60.6
2FY6	1.85	1Z5Y	0.184	2.037	24.5	0.544	0.544	81.7	81.7	80.9
4M8J	3.29	4C7R	0.261	2.038	126.1	0.472	0.464	65.5	57.9	45.5
4WPL	1.15	4LZB	0.198	2.041	28.5	0.548	0.548	87.5	87.3	80.9
5B43	2.80	5MGA	0.390	2.041	228.5	0.558	0.548	68.1	63.0	61.8
4BRU	3.25	6B4J	0.338	2.046	88.3	0.528	0.506	56.3	51.9	56.3
5IXP	1.73	6Q5G	0.169	2.047	50.8	0.534	0.534	80.0	79.4	76.4
5BPW	3.40	5BPT	0.630	2.048	278.5	0.516	0.500	57.9	55.0	43.5
6APE	1.45	1EE9	0.230	2.049	35.2	0.553	0.552	84.2	84.0	50.5
3MTT	3.30	3AJW	0.122	2.056	120.0	0.505	0.502	85.3	85.5	85.3
4X9Y	1.07	1E3Z	0.189	2.061	36.7	0.549	0.548	86.2	86.0	85.2
3B2S	1.92	4G2M	0.196	2.066	36.8	0.577	0.577	81.7	80.0	68.8
4W1S	2.30	1UKC	0.257	2.066	44.2	0.475	0.489	73.0	69.2	61.5
5OQ3	1.35	3FND	0.119	2.066	29.0	0.545	0.545	87.4	86.3	86.1
6BY9	2.30	5D66	0.243	2.068	61.8	0.539	0.524	73.8	70.4	71.2
5Z5D	1.70	3ZXK	0.245	2.088	45.3	0.540	0.540	85.7	82.9	74.4
6DYS	2.30	2QEC	0.132	2.098	28.0	0.525	0.522	81.3	81.2	81.2
4B65	2.32	5O8P	0.253	2.112	44.9	0.546	0.545	76.7	72.7	67.7
4EC3	2.65	6F72	0.188	2.121	32.5	0.522	0.531	78.0	77.8	74.5
5T39	1.10	1Y8C	0.125	2.124	21.8	0.556	0.555	87.5	87.2	87.9
6M8Y	1.10	4I0W	0.170	2.130	43.4	0.540	0.530	68.7	63.0	68.1
5XBX	1.04	3X2M	0.288	2.134	82.2	0.557	0.512	50.4	50.4	49.3
6AJK	3.00	1QAM	0.204	2.137	30.5	0.562	0.578	80.0	79.5	77.0
6EWE	1.46	6R9N	0.163	2.138	27.3	0.534	0.521	86.1	86.2	84.1
4HTV	3.00	1XM9	0.155	2.146	66.1	0.526	0.518	73.1	63.2	59.4
5T4C	1.80	5XC2	0.214	2.148	76.9	0.549	0.547	83.3	83.1	77.8
2YN0	1.50	3HJG	0.208	2.165	47.6	0.551	0.547	76.5	75.9	70.7
4L9M	3.00	6AXF	0.469	2.171	73.2	0.510	0.542	75.5	71.3	60.6
4WE8	2.10	4FIU	0.357	2.185	64.6	0.568	0.553	73.0	45.4	45.7
3MDU	1.40	2OOD	0.169	2.189	37.9	0.579	0.579	87.5	87.6	86.0
4CZK	2.60	5NBM	0.170	2.197	25.5	0.537	0.547	82.3	81.9	75.2
2BHA	2.40	5FCF	0.251	2.209	109.1	0.563	0.552	64.7	57.4	62.6
2WDF	2.10	4H2I	0.211	2.220	44.6	0.551	0.544	81.7	81.0	76.2
1O1Y	1.70	2VDJ	0.125	2.225	34.4	0.562	0.567	83.8	83.3	81.6
3L0P	3.00	1TEV	0.256	2.230	35.7	0.552	0.542	79.1	78.9	73.4
4YRS	2.75	1Z7M	0.198	2.248	32.8	0.523	0.528	79.0	80.2	78.2
5O71	3.28	6H4I	0.594	2.248	214.0	0.545	0.487	51.5	49.9	48.4
5CE6	1.70	4ZNG	0.151	2.276	51.8	0.548	0.537	72.3	63.6	56.3
6F47	1.35	6RIM	0.216	2.316	64.0	0.526	0.529	79.5	66.2	80.2
4IKX	2.30	5A2N	0.188	2.319	109.6	0.481	0.491	85.1	84.9	84.4
4DU4	2.28	4QCL	0.170	2.334	54.0	0.554	0.561	81.0	80.6	70.4
5YDM	2.50	4AMN	0.273	2.340	59.9	0.576	0.554	77.6	76.5	61.1
6Q7O	2.00	3I76	0.168	2.381	20.7	0.572	0.578	85.9	86.4	87.4
5MW5	2.70	4XBM	0.452	2.543	54.9	0.506	0.502	66.2	64.6	62.4
5F4V	2.90	5B5K	0.545	2.549	22.0	0.517	0.491	70.5	71.2	67.4
5CVR	2.60	5I2D	0.166	2.563	38.3	0.515	0.532	70.0	67.3	67.2
5W17	2.58	1QJ9	0.061	2.572	35.7	0.569	0.554	83.7	83.7	84.0

Target structure	Target resolution	Search structure	Sequence identity	GESAMT RMSD	PHASER LLG	Initial R-free	Refined R-free	Phase error CR	JB	SF
2XO7	2.85	5DKU	0.251	2.640	60.7	0.536	0.535	77.8	77.8	71.0
5YF8	3.40	3NAH	0.119	2.661	40.9	0.564	0.569	80.7	79.5	77.3
3K5Y	2.30	5YKI	0.240	2.693	36.2	0.545	0.547	70.8	70.2	64.8
2XZP	2.72	2XZL	0.526	2.737	131.0	0.530	0.525	59.8	54.5	59.6
4IFQ	3.25	4KNH	0.186	2.773	73.5	0.559	0.558	73.2	65.8	58.6
5ER3	2.10	3C6Q	0.139	2.805	49.7	0.538	0.532	69.9	66.1	61.0
3LGC	2.77	1V98	0.093	2.821	22.8	0.546	0.577	79.9	80.8	80.7

Table S1: PDB structures and search models used in testing. Resolution is the resolution of the observed data as deposited at the PDB. GESAMT sequence identity is a proportional similarity between the sequences. GESAMT RMSD is a measure of conformational similarity between conserved residues. The PHASER LLG is a standard measure of the correctness of a molecular replacement solution. R factors are for the PHASER-positioned and editted model before and after preliminary refinement in Refmac5. Mean phase errors are given for models refined by conventional refinement (CR), jelly body (JB) and shift field (SF).

	R-work	R-free
Sequence identity	-0.6570	-0.6367
GESAMT RMSD	0.6813	0.7141
Phaser LLG	-0.8197	-0.8259
Initial R-work	0.6787	0.6844
Refined phase error	0.9092	0.9259

Table S2: Comparison of refined R-work and R-free to various structure metrics. The Spearman rank correlation coefficient is used due to non-linearity. Refined R-free is a better intrinsic predictor than refined R-work of refined phase error due to the structure-dependent bias in R-work, as expected from Lunin and Skovoroda (1995). R-free also shows higher correlation with all of the other metrics except for sequence identify, including with the pre-refinement R-work.

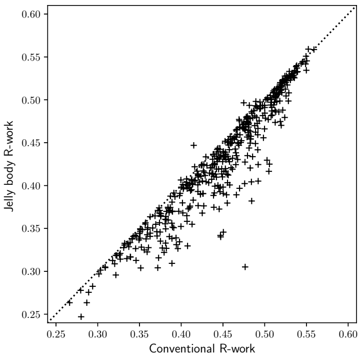

(a)

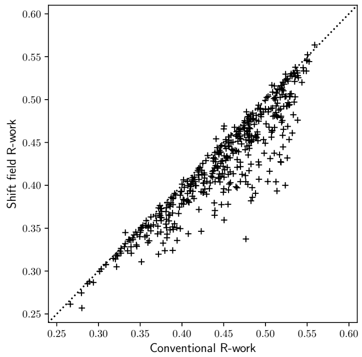

(b)

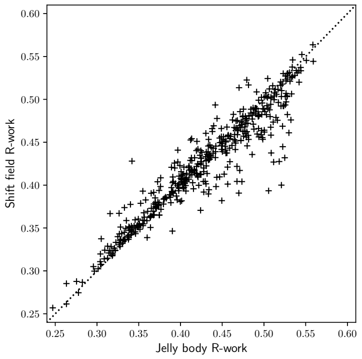

(c)

Figure S1: Comparison of crystallographic (work) R factor after the different refinement procedures: (a) Jelly body compared to conventional refinement alone, (b) Shift field compared to conventional refinement alone and (c) Shift field compared to Jelly body refinement. Points below the diagonal indicate a better result for the method on the y-axis.

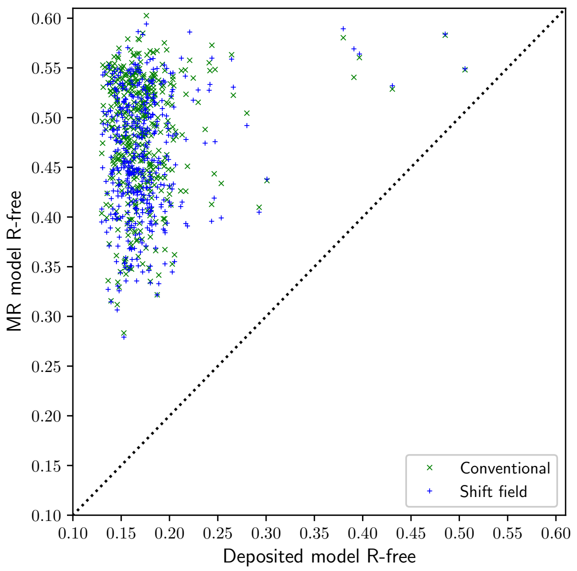

Figure S2: Comparison of free R factor after conventional or shift field refinement of the MR model to the free R factor of the conventionally refined deposited model. The MR models are substantially incomplete as a result of the homology selection criteria used in preparation of the test set.

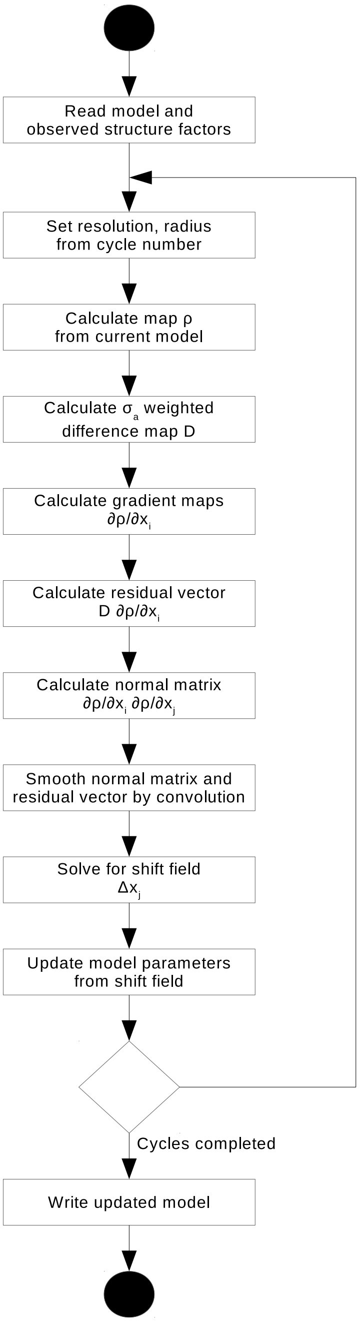

Figure S3: Process diagram for the shift field calculation (slightly simplified compared to the description in the main text.)
